# Deep mineral water accelerates recovery after dehydrating aerobic exercise: a randomized, double-blind, placebo-controlled crossover study

**DOI:** 10.1186/1550-2783-11-34

**Published:** 2014-06-26

**Authors:** Loreta Stasiule, Sandrija Capkauskiene, Daiva Vizbaraite, Arvydas Stasiulis

**Affiliations:** 1 Lithuanian Sports University, Kaunas, Lithuania

**Keywords:** Deep mineral water, Rehydration, Aerobic capacity, Leg muscle power, Recovery, Women

## Abstract

**Background:**

The effect of deep mineral water (DMW) with moderate mineralization on the recovery of physical performance after prolonged dehydrating aerobic exercise in the heat was studied in nine healthy, physically active (VO_2_max = 45.8 ± 8.4 mL kg^−1^ min^−1^) women aged 24.0 ± 3.7 years.

**Methods:**

We conducted a randomized, double-blind, placebo-controlled crossover human study to evaluate the effect of ingestion of natural mineral water extracted from a depth of 689 m on recovery from prolonged fatiguing aerobic running conducted at 30°C.

**Results:**

Mean body weight decreased by 2.6–2.8% following dehydrating exercise. VO_2_max was 9% higher after 4 h of recovery after rehydrating with DMW compared with plain water. Leg muscle power recovered better during the slow phase of recovery and was significantly higher after 48 h of recovery after rehydrating with DMW compared with plain water.

**Conclusions:**

DMW with moderate mineralization was more effective in inducing recovery of aerobic capacity and leg muscle power compared with plain water following prolonged dehydrating aerobic running exercise.

## Background

Prolonged exercise performed at high temperature increases metabolic rate and heat production [1], and causes dehydration [2]. Even modest (up to 2% of body weight) exercise-induced dehydration attenuates aerobic performance [3] and impairs cognitive function [4,5]. Athletes often train or compete on consecutive days or more than once per day and must consume sufficient fluid to restore water balance or to replace fluid losses before the next exercise session. A fluid deficit incurred during one exercise session may compromise performance in the next exercise session if fluid replacement is insufficient [6]. Fluid intake can attenuate or prevent many of the disturbances in metabolic, cardiovascular, thermoregulatory functions, and performance that accompany dehydration [7-9]. Therefore, it is important to replace fluid and electrolytes rapidly to recover fully before the start of the next bout of exercise [10,11]. This is particularly challenging when sweat loss is high and the interval between exercise bouts is short. Both the volume of the rehydration fluid and its composition are critical for maintaining whole-body fluid homeostasis [12].

More than 3,000 brands of mineral water are commercially available worldwide [13]. Several studies have evaluated the effects of ingestion of water or commercially available drinks on the restoration of fluid balance after exercise-induced dehydration [14-19]. Only a few studies have evaluated the effects of natural and widely used mineral waters on restoration of performance after dehydrating exercise [16,19-21]. It has been shown recently that desalinated ocean mineral water, taken from 662 m below sea level, can substantially accelerate recovery of aerobic power and lower-body muscle power after a prolonged bout of dehydrating exercise [21].

Natural deep mineral water of moderate mineralization (DMW) is extracted from a depth of about 700 m in geological sandstone, dolomite, and gypsum layers, which were formed almost 400 million years ago. The DMW in these layers is 10,000–13,000 years old. The composition of this calcium–magnesium–sulfate water was conditioned by a complex metamorphosis that took place in the ground and that involved the melting of calcium and magnesium minerals contained in the dolomite and gypsum layers. Presently, there is no information about the effects of DMW on recovery after exercise performed in a warm environment causing dehydration. We propose that the unique composition of DMW with moderate mineralization may accelerate the recovery in such exercise conditions. To test this hypothesis, we conducted a human study in which we compared the short-term recovery of physical performance after a prolonged, aerobic dehydrating exercise (ADE) in subjects given DMW or placebo (plain water) drink supplied for rehydration.

## Methods

### Subjects

Nine healthy, physically active, nonsmoking women (age 24.0 ± 3.7 years; height 172.7 ± 7.3 cm; weight 69.0 ± 9.9 kg; body fat 21.2% ± 4.4%) who were not taking any medications volunteered to participate in the study. During the first meeting, the subjects were introduced to the objectives and study design, and were instructed about dietary and rest regimens. The subjects were asked not to perform any other physical activities during recovery period and on the eve of testing they were instructed to maintain their accustomed dietary and drinking regimen during both trials. To achieve this 72-h food diary was completed during the first trial, and subjects were asked to follow the same diet during the second trial. Written informed consent was obtained after explanation of the purpose and experimental procedures of the study. Aerobic capacity was measured during a continuous incremental treadmill test until exhaustion no less than 5 days before (maximal oxygen uptake (VO_2_max) 45.8 ± 8.4 mL kg^−1^ min^−1^). The subjects consumed randomly selected water without being informed about the type of water. The research protocol was approved by the Human Research Ethics Committee.

### Drinks

The concentrations of the minerals and trace elements of the drinks used are shown in Table 1. The DMW with moderate mineralization was used in this study. Purified tap water was used as the placebo drink.

**Table 1:** Concentrations of the minerals and trace elements in drinks used in the study

**Mineral**	**Placebo (mg L**^**−1**^**)**	**DMW (mg L**^**−1**^**)**
Na	5.6	76
K	0.8	19
Ca	20.7	220
Mg	9.7	73
Cl	12.5	46
SO_4_	12.9	834
F	0.08	0.3
**Trace element**	**Placebo (mg L**^**−1**^**)**	**DMW (mg L**^**−1**^**)**
Cu	0.003	0.0054
Fe	< 0.001	1.2326
Mn	0.009	0.0163
Cr	< 0.006	0.0025
P	N. D.	0.5434
B	N. D.	0.4175
Zn	N. D.	0.0124

### Physical performance

Aerobic power (VO_2_max) and peak lower-body muscle power were the physical performance measures selected for assessing the degree of recovery. VO_2_max was measured using the ramp exercise test. This test comprised a 4-min warm-up followed by an incremental continuous increase in speed of 0.1 km/h every 6 s until volitional fatigue. The criteria used to verify that VO_2_max was achieved were a respiratory exchange ratio greater than 1.1, maximum heart rate equal to 220 – age ± 10 beats per min, and a plateau in oxygen uptake with increasing workload (all the criteria had to be met). Pulmonary gas exchange was analyzed using a portable analyzer (Oxycon Mobile; Jaeger, Hoechberg, Germany). Before each test, the equipment was calibrated according to the manufacturer’s recommendations.

Peak lower-body muscle power was assessed using a multicomponent Kistler force plate (type 9286A, USA). Each subject performed three repetitions of maximal counter-movement jumps from a 90° knee flexion to full extension keeping the hands on the hips. There was a 1 min rest between jumps. Vertical jump height was calculated using the formula of Bosco et al. [22]: h = Ft^2^ × 1.226, where h = jump height (m) and Ft = flight time (s). The values of the two best jumps were averaged and used in the statistical analysis.

### Biochemical analysis

To measure plasma creatine kinase (CK) activity, 0.5 mL of capillary blood was taken from a finger using collection tubes and then analyzed with an automatic biochemical analyzer (Spotchem II, Japan). After 5 min of recovery from the ramp exercise test, capillary blood was collected to measure lactate concentration using an Accutrend lactate analyzer (Germany).

### Experimental design

ADE similar to that used by Hou et al. was used in this study [21]. The subject was asked to run on a motorized treadmill at 40% of VO_2_max at a room temperature of 30°C until a 3% decline in body mass was observed; the average running speed was 8.1 ± 1.9 km h^−1^, and the average running time was 96.7 ± 19.4 min. During recovery, the subject consumed pure water or DMW at an amount equivalent to 1.5 times her body mass loss [23]. Water supplements were evenly divided into five equal parts and were ingested at 30 min intervals. Measures of physical performance (aerobic power and lower-body muscle power) and blood CK activity were assessed at 4, 24, and 48 h during the recovery period. To control for possible confounding effects of individual variation, a randomized, double-blind crossover design was used with trials spaced 7 days apart.

### Statistical analysis

All values are expressed as the percent of baseline (mean ± standard deviation). Two-way analysis of variance with repeated measures was used to compare between DMW and pure water trials at specified time points during recovery. A paired *t* test with Bonferroni’s correction was used to compare treatment differences at each time point. Probability of a type I error less than 5% was considered statistically significant.

## Results

The concentrations of the minerals and trace elements in the drinks are shown in Table 1.

ADE decreased body weight by 2.6–3.1%. Body weight increased significantly during recovery compared with the value immediately after exercise but remained significantly lower than before ADE. Body weight did not differ significantly between trials (Table 2).

**Table 2:** Changes in body weight

	**Before ADE**	**After ADE**	**Weight lost%**	**After 4 h**	**After 24 h**	**After 48 h**
DMW	69.3 (10.4)	67.4 (10.1)	2.8 (0.2)	68.6 (10.4)*^#^	68.5 (10.1)*^#^	68.8 (10.1)*
Placebo	69.5 (11.6)	67.6 (11.3)	2.8 (0.2)	68.7 (10.4)*^#^	68.5 (9.9)*^#^	68.6 (9.9)*^#^

*Significant difference (p < 0.05) compared with after ADE; ^#^significant difference (p < 0.05) compared with before ADE.

**Figure 1:**
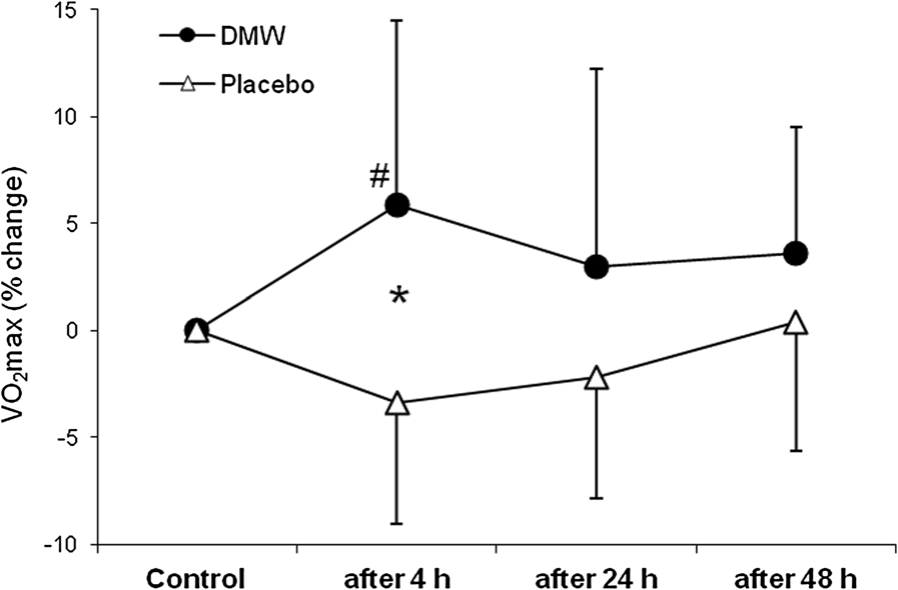
**Changes in maximum oxygen uptake during recovery. **^#^p < 0.05 compared with control in the DMW condition; *p < 0.05 for the comparison between placebo and DMW.

**Figure 2:**
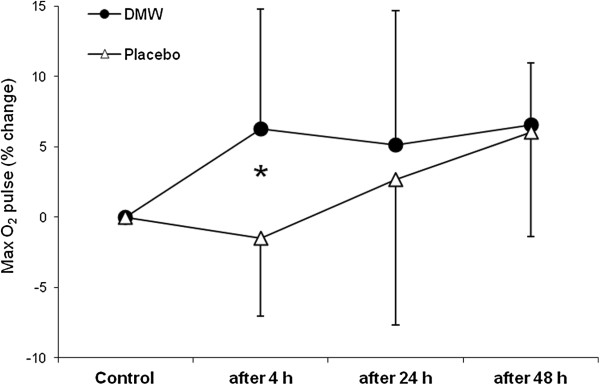
**Changes in maximum oxygen pulse during recovery.** *p < 0.05 between DMW and placebo trials.

**Figure 3:**
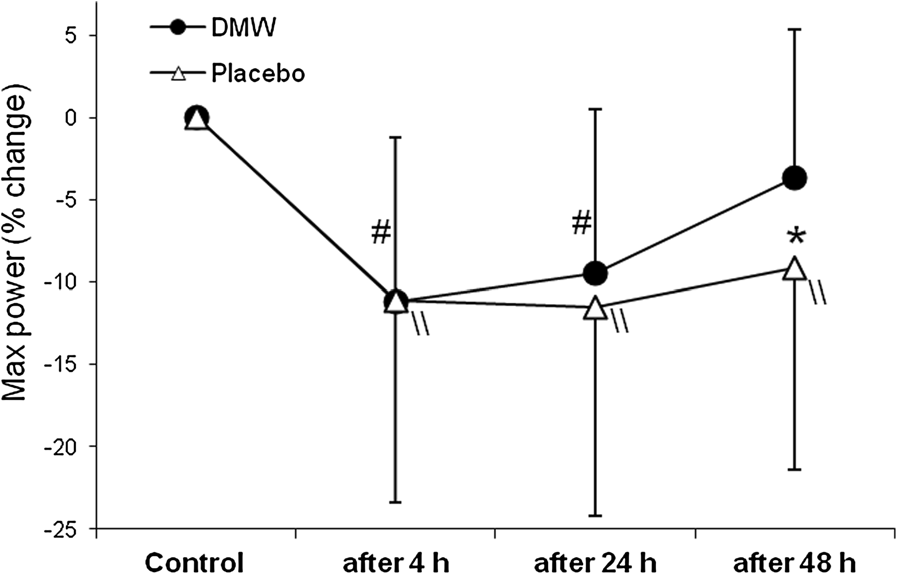
**Changes in vertical jump height during recovery. **^#^p < 0.05 during recovery in the DMW trial compared with control; ^\\^p < 0.05 during recovery in the placebo trial compared with control; *p < 0.05 between the DMW and placebo trials.

**Figure 4:**
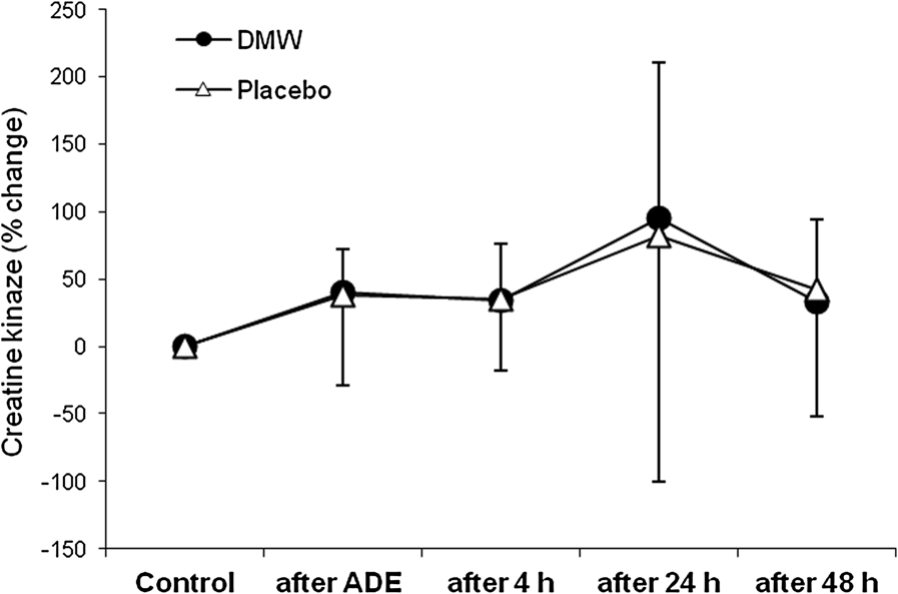
Changes in the activity of plasma creatine kinase during recovery.

In the placebo condition, VO_2_max was slightly (2.4%) lower 4 h after ADE than control value (Figure 1). By contrast, VO_2_max increased at this time in the DMW condition and was significantly higher by 9% compared with the placebo trial (effect size – 1.26).In the DMW trial, peak oxygen pulse was significantly higher by 5.4% at 4 h of recovery compared with control and by 7.5% compared with the placebo trial (Figure 2).Jump height was significantly reduced by ~11% in both trials (p < 0.05). Jump height returned to the control level 48 h after ADE in the DMW trial and was significantly higher (by ~6.6%, effect size – 0.52) than in the placebo trial at this time (Figure 3).CK activity showed a tendency to increase 24 h after ADE in both trials, but the differences were not significant between trials or compared with control (p > 0.05) (Figure 4).

## Discussion

In this study, we found that DMW with moderate mineralization extracted from a well at a depth of 689 m accelerated the short-term recovery of aerobic power and lower-body muscle power after a prolonged bout of dehydrating exercise in the heat.

We focused only on performance after rehydration with DMW or placebo and compared the recovery of these parameters 4, 24, and 48 h after dehydrating exercise in the heat. Thus, we do not have data on the extent to which performance was reduced in the hypohydrated state immediately after the ADE. Based on the literature, even modest exercise-induced dehydration of up to 2% of body weight can attenuate aerobic capacity [3,6]. Another study reported only a small decrease in VO_2_max but a larger decrease in graded exercise time 1 h after dehydrating exercise causing a loss in body weight of 1.8–2.1% [19]. The subjects in our study lost nearly 3% of body weight after ADE, and one could expect a greater impact on performance than in the reports cited above.

Replacement of sweat loss should help restore exercise capacity when the impairment is a consequence of a body water deficit. The type and amount of fluids ingested in the recovery period after exercise can significantly influence the restoration of fluid balance [10]. Full recovery of fluid balance can be achieved only when a significant, albeit insufficient, quantity of sodium is ingested after exercise. It has been shown that addition of 40–50 mmol/L–1 of sodium chloride to a rehydration beverage reduced subsequent urine output, thereby providing more effective rehydration than a sodium-free drink. However, this did not result in improved performance 4 h after the end of the rehydration period [20]. Thus, the low concentration of sodium in DMW would not have slowed recovery of performance in our study.The concentration of sodium in both drinks used in our study was low, and it seems that 4 h after ADE, the subjects were slightly dehydrated. The body weight was lower by 0.4–0.7 kg or 0.6–1.0% compared with before ADE but there was no significant difference between trials.

There is a limited range of commercially available mineral waters that have a composition sufficient to achieve full rehydration, even though it is generally thought by the public that some well-known drinks are effective for this purpose [15]. In the study by Shirreffs et al., volunteers were dehydrated by 1.94 ± 0.17% of body mass after intermittent exercise in the heat and then ingested a carbohydrate–electrolyte solution (Gatorade), carbonated water/apple juice mixture (Apfelschorle), or San Benedetto mineral water in a volume equal to 150% of the loss of body mass, and the responses were compared with the rehydration effectiveness of Evian mineral water. Four hours after rehydration, the subjects were in a significantly lower hydration status than the pretrial situation in the trials with Apfelschorle (−365 ± 319 mL, p = 0.030), Evian (−529 ± 319 mL, p < 0.0005), and San Benedetto (−401 ± 353 mL, p = 0.016) but were in the same hydration status as before the dehydrating exercise in the trial with Gatorade (−201 ± 388 mL, p = 0.549) [14]. Thus, water ingestion results in prompt diuresis, even during hypohydration, and prevents a return to the normal hydration state [24,25].

Despite the use of commercially available solutions and mineral waters to assess their influence on rehydration and recovery of performance in several studies, it is difficult to compare the data because of differences in the magnitude of dehydration and study designs. In the study by Snell et al. [19] the subjects exercised at 70-75% VO_2_max for 60 min at 29-33°C, resulting in a dehydration weight loss of 1.8-2.1% body weight. After 60 min of rest, subjects performed treadmill test to voluntary exhaustion, which resulted in a small reduction in VO_2_max and a decline in treadmill performance by 3% relative to the control results. During next 60 min of rest subjects ingested the same amount of fluid lost in the form of one of three randomly assigned commercial drinks and then repeated the treadmill test to voluntary exhaustion. VO_2_max returned to baseline levels with Rehydrate, but there was only a slight improvement with Gatorade and Crystal Light. There were no differences in heart rate or ventilation with the three different replacement drinks. Relative to the dehydrated state, a 6.5% decrease in treadmill performance time occurred with Crystal Light (flavored water product), while replenishment with Gatorade, which contains fructose, glucose, sodium, and potassium, caused only a 2.1% decrease. By contrast, Rehydrate, which contains fructose, glucose polymer, calcium, magnesium, sodium, potassium, amino acids, thiols, and vitamins, caused a 7.3% increase in treadmill time relative to the dehydrated state [19]. Kalman et al. [16] compared the effects of ingestion of supermarket brand bottled water, pure coconut water, coconut water from concentrate, or a carbohydrate–electrolyte sport drink (5–6% carbohydrate solution). They found that all were capable of promoting rehydration 1 h after dehydrating exercise and that treadmill performance during the rehydration period did not differ between drinks. Subjects lost ~1.7 kg (~2% of body mass) during the dehydrating exercise. Addition of 40 or 50 mmol/L of sodium chloride to a rehydration beverage reduced subsequent urine output, thereby providing more effective rehydration than a sodium-free drink; however, this did not improve performance 4 h after the end of the rehydration period [20]. Similar to our study, another recent study reported that desalinated ocean mineral water taken from 662 m below sea level substantially accelerated recovery in aerobic power and increased lower-body muscle power after a prolonged bout of dehydrating exercise [21]. The physical challenge protocol in that study induced a prolonged impairment of aerobic power (more than 10%) that was present for 48 h during recovery with purified water. We applied a similar physical challenge but found a smaller difference in VO_2_max between conditions (9%) 4 h after ADE. There is no obvious explanation for this difference. The VO_2_max values were similar in both studies: 45.8 in our study and 49.7 mL kg^−1^ min^−1^ in the study by Hou et al. [21]. Our participants were female physically active students, and their aerobic capacity may be considered higher because of the 10–15% difference between female and male untrained persons, as well as athletes, because of morphophysiological differences [26]. VO_2_max at the same fat-free mass is considerably (~30%) higher in sedentary men than in sedentary women [27]. Mild hypohydration exacerbates cardiovascular and thermoregulatory strain and tends to impair endurance performance, but greater aerobic fitness attenuates these physiological effects. However, in a study by Merry et al. [28], performance power was reduced by 13% in untrained subjects and by 7% in trained subjects without an effect of fitness (p = 0.38). The effects of hyperthermia on VO_2_max and physical performance in men and women are almost identical [29]. Women seem not to be disadvantaged when there is rapid and complete restoration of exercise-induced sweat loss. In the study by Maughan et al. [30], five women with a regular menstrual cycle exercised in the heat to dehydrate themselves by 1.8% of body mass at three different stages of their menstrual cycle (2 days before, and 5 and 19 days after the onset of menses). Beginning 30 min after exercise and extending for > 60 min, they consumed the same quantity of the same beverage on each occasion. The acute replacement of volume loss incurred by sweat loss after exercising in the heat did not differ between different states of the menstrual cycle.

The question arises as to the reason for better VO_2_max recovery in our study when rehydrating with DMW. The maximum oxygen pulse changed in a similar manner as VO_2_max, but at 4 h of recovery it was 7% higher in the DMW trial. The oxygen pulse is significantly related to stroke volume but not to the arteriovenous O_2_ difference in men and women [31]. One possible explanation is that stroke volume recovered better in the DMW trial and that this led to a faster and better recovery of VO_2_max. In humans, VO_2_max is limited by the ability of the cardiorespiratory system to deliver oxygen to the exercising muscles [32]. It has been established recently that maximum heart rate and myocardial work capacity do not limit VO_2_max in healthy individuals [33]. Munch et al. [33] found that limited left ventricular filling and possibly altered contractility reduce stroke volume during atrial pacing, whereas a plateau in left ventricular filling pressure appears to restrict cardiac output close to VO_2_max. The left ventricular filling may be associated with blood plasma volume. Experiments with plasma volume expansion showed that 200–300 mL of plasma volume expansion increased stroke volume measured during submaximal exercise and, consequently, increased VO_2_max and performance in untrained men [34]. Expansion of the plasma volume is a well-recognized early response to endurance training and is observed even as an acute response to a single bout of intense exercise. The onset of the phenomenon is extremely rapid: hypervolemia is observed within minutes or hours of the cessation of exercise. However, 2 days are necessary to reach peak plasma volume expansion after a marathon or ultramarathon run. The magnitude of this natural expansion ranges from 9% to 25%, corresponding to an additional 300–700 mL of plasma. Hypervolemia can improve performance by inducing better muscle perfusion and by increasing stroke volume and maximal cardiac output. By increasing skin blood flow, plasma volume expansion also enhances thermoregulatory responses to exercise [35]. The effects of plasma volume expansion or training on stroke volume or VO_2_max do not differ between men and women [36]. Thus we suppose that this parameter recovered better in DMW trial ensuring better recovery of stroke volume and VO_2_max.

In our study, muscle power remained significantly reduced in the placebo trial but recovered faster and approached the control level 48 h after ADE in the DMW trial. CK activity changed in a similar manner in both trials and was elevated 24 h after ADE. Decreased muscle power and elevated CK activity indicate the presence of fatigue, which may be associated with muscle damage. Warren et al. [37] suggested that measures of muscle function such as strength and power are effective indicators of both the magnitude and time course of muscle damage. The exercise conditions that can induce muscle damage are unaccustomed exercise and exercise with higher intensity or longer duration than those to which the subject is adapted [38,39]. Because a high number of concentric and, particularly, eccentric contractions are performed during long-distance running, the symptoms of muscle damage are usually observed immediately and a few days after a running bout even in experienced runners [40]. Our participants included running exercise in their daily regular physical activity, and this may explain the modest increase in CK activity compared to Hou et al. [21] data. An unaccustomed running duration may be the main reason for changes in CK activity and muscle power in our participants.

The key components of DMW contributing to the observed ergogenic benefits are not known. In our study, the calcium–magnesium–sulfate DMW was taken from a depth of about 700 m and is characterized by enriched contents of boron, phosphorus, chromium, manganese, iron, and copper. Hou et al. [21] speculated that the effect of deep ocean water on accelerating recovery after fatigue may be associated with the attenuation of exercise-induced muscle damage. It has been found that the main supplements that seem to protect against muscle damage are the flavonoids, which are known for their efficient anti-inflammatory and antioxidant properties [41]. Howatson et al. [42] reported that runners who consumed tart cherry juice for 5 days before and 48 h after a marathon showed faster recovery of muscle strength and reduced inflammation [42].

However DMW used in our study as well as deep ocean water do not contain such components. Possibly the minerals and trace elements in DMW may work cooperatively to restore normal human performance. Snell et al. [19] reported that recovery was significantly faster when consuming a rehydration drink containing fructose, glucose polymer, calcium, magnesium, sodium, potassium, amino acids, thiols, and vitamins compared with Crystal Light, while replenishment with Gatorade, which contains fructose, glucose, sodium and potassium [20]. It is possible that the different effects on performance between a rehydration drink and Gatorade may be associated with higher concentration of calcium and magnesium in the rehydration drink. This may explain the better recovery of performance in our study in the DMW trial because DMW is rich in calcium and magnesium. In animals, a lack of dietary magnesium leads to increased free radical production [43], and magnesium supplementation eliminates free radical production induced by ischemia– reperfusion [44] and alcohol consumption [45]. Serum magnesium concentration and dietary magnesium intake are known correlates of muscle strength [46,47]. It has been recently shown that magnesium enhances glucose availability in the peripheral and central systems and increases lactates clearance in the muscle during exercise in rats [48]. Hou et al. [21] also speculated that a higher content of magnesium, lithium, and rubidium in deep ocean mineral water might be associated with strengthened antioxidant capability against oxidative stress during postexercise recovery [44,49,50]. In DMW, the content of boron (0.417 mg/L), which is now considered an essential nutrient for humans, is twice that found in human serum (~0.2–0.3 mg/L) [51]. Boron is known to attenuate the exercise-induced rise in plasma lactate concentration in animals [52] and to prevent magnesium loss in humans [53].

On the application side, we have demonstrated for the first time the benefit of acute DMW supplementation on recovery of performance after prolonged ADE in a warm environment. An imbalance between the loss and gain of essential minerals and trace elements after prolonged exercise in the heat may delay recovery.

## Conclusions

Ingestion of DMW accelerated recovery of aerobic capacity and leg muscle power compared with ingestion of water alone. This might reflect increased restoration of cardiac capacity and attenuation of the indicators of muscle fatigue or damage.

## Competing interests

The authors declare that they have no competing interests.

## Authors’ contributions

LS, SC, DV and AS designed the experiments. LS, SC and DV performed the experiments. LS and AS performed the statistical analyses. AS, LS and DV wrote the manuscript. All the authors read and approved the final manuscript.

## Authors’ information

All the authors are from Department of Applied Biology and Rehabilitation, Lithuanian Sport University, Kaunas, Lithuania.
